# Supplementary material for: Comparison of Two Contemporary Quantitative Atherosclerotic Plaque Assessment Tools for Coronary Computed Tomography Angiography: Single-Center Analysis and Multi-Center Patient Cohort Validation
Source: Diagnostics (Basel). 2024 Jan 9;14(2):154. doi: 10.3390/diagnostics14020154 (PMC10814854; doi:10.3390/diagnostics14020154)
Supplement: Supplementary file 1 [file diagnostics-14-00154-s001.zip › diagnostics-2791912-supplementary.pdf]

Suppl. Table S1. CT scanner, technical parameters and CCTA image data.

Scanner Typ	Cohort #1	Slice Thickness (average)	Slices	table feed [mm/s]	max pitch	minimal gantry rotation time [ms]	Temporal resolution [ms]	tube voltage [kV range]	tube current [mA range]
Siemens, Somatom Force	All	0.58	384	737	3.2	250	66	70-150	290-560

Scanner Typ	Cohort #2	Slice Thickness (average)	Slices	table feed [mm/s]	max pitch	minimal gantry rotation time [ms]	Temporal resolution [ms]	tube voltage [kV range]	tube current [mA range]
Siemens, Somatom Definition AS+	2 (4.0%)	0.69	128	192	1.5	300	150	70-140	20-800
Siemens, Somatom Definition Flash	7 (14.0%)	0.73	256	430	3.4	280	75	70-140	250-500
Siemens, Somatom Force	32 (64.0%)	0.58	384	737	3.2	250	66	70-150	290-560
Philips, Brilliance CT 6000 (iCT)	4 (8.0%)	0.8	256	185	1.8	270	135	80-140	10-1000
GE Healthcare, Revolution CT	5 (10.0%)	0.63	256	300	1.5	280	140	70-140	1200

Suppl. Table S2. Data acquisition protocols and the median heart rate during CCTA, contrast dosage and radiation exposure.

	All Patients		Low-pitch retrospective acquisition		Axial acquisition in diastole[1]		High-pitch prospective acquisition (flash)		Axial acquisition in systole[2]		Other	
	#1	#2	#1	#2	#1	#2	#1	#2	#1	#2	#1	#2
	n = 50	n = 50	n = 30 (60.0%)	n = 6 (12.0%)	n = 17 (34.0%)	n = 18 (36.0%)	n = 0 (0.0%)	n = 11 (22.0%)	n = 1 (2.0%)	n = 15 (30.0%)	n = 2 (4.0%)	n = 0 (0.0%)
Median contrast agent dosage (mL)	75.7 (50.0-97.0) (6%)*	72.1 (22.0-202.0)	77.8 (70.0-80.0) (7%)*	68.3 (60.0-70.0)	70.0 (50.0-97.0) (6%)*	68.1 (55.0-100.0)	0.0	61.1 (22.0-70.0)	80.0 (80.0-80.0)	84.8 (60.0-202.0)	90.0 (90.0-90.0)	0.0
Dose length product (mGy*cm)	471.5 (42.0-1845.4) (2%)*	230.4 (24.0-849.0)	571.9 (42.0-1845.4) (3%)*	413.3 (163.0-849.0)	262.4 (93.0-671.0)	171.9 (57.0-314.0)	0.0	48.6 (24.0-91.0)	180.0 (180.0-180.0)	286.3 (77.0-660.0)	938.0 (828.0-1048.0)	0.0
Median heart rate	60.8 (44.0-90.0) (24%)*	68.6 (50.0-110.0)	61,9 (50.0-82.0) (30%)*	67.7 (50.0-75.0)	57.6 (45.0-75.0) (17%)*	66.7 (55.0-75.0)	0.0	58.8 (50.0-80.0)	90.0 (90.0-90.0)	78.3 (60.0-110.0)	57.0 (52.0-62.0)	0.0
Median Tube voltage (kV)	101.5 (70.0-120.0) (48%)*	0.0	86.0 (70.0-120.0) (66%)*	0.0	111.3 (100.0-120.0) (6%)*	0.0	0.0	0.0	0.0	0.0	0.0	0.0
Beta-blockers administration necessary	33 (20%)*	39	19 (30%)*	5	12	15	0	9	1	10	1 (50%)*	0

#1 indicates Cohort 1 and #2 indicates Cohort 2
* patients [%] with missing values.
1 padding 60-80%, 2 padding 250-450ms
